# Using stable isotopes to analyse extinction risks and reintroduction opportunities of native species in invaded ecosystems

**DOI:** 10.1038/s41598-020-78328-9

**Published:** 2020-12-10

**Authors:** Phillip J. Haubrock, Paride Balzani, J. Robert Britton, Peter Haase

**Affiliations:** 1 Department of River Ecology and Conservation, Senckenberg Research Institute and Natural History Museum Frankfurt, Clamecystrasse 12, 63571 Gelnhausen, Germany; 2 Faculty of Fisheries and Protection of Waters, CENAKVA, University of South Bohemia in České Budějovice, Vodňany, Czechia; 3 Department of Biology, University of Florence, Via Madonna del Piano 6, 50019 Florence, Italy; 4 Department of Life and Environmental Sciences, Faculty of Science and Technology, Bournemouth University, Poole, Dorset UK; 5 Faculty of Biology, University of Duisburg-Essen, 45141 Essen, Germany

**Keywords:** Community ecology, Freshwater ecology, Invasive species

## Abstract

Invasive non-native species have pervasive impacts on native biodiversity, including population extirpations and species extinctions. Identifying reasons why a population of a native species is extirpated following an invasion often relies on literature-based results of anecdotal observations. The well-established schemes of existing risk assessments for invasive species assume that a species’ information (e.g. impacts or behavioural and biological traits) can be projected from one area to another to estimate the potential impact of a species in another environment. We used stable isotope data (δ^13^C, δ^15^N) from both invaded and uninvaded communities to predict such invasion impacts by reconstructing trophic relationships. This approach was tested on a community within a protected lake in Northern Spain where, following the introductions of non-native species, the last resident native species (the common tench *Tinca tinca*, the European eel *Anguilla anguilla*, and the whirligig beetle *Gyrinus* sp.) had been extirpated. Through the application of this novel approach, we found evidence that native species’ declines were related to direct predation by and resource overlap with non-native species, which occurred in conjunction with habitat modification. Using this approach, we outlined the mechanisms involved in the extirpation of native species in the post-invasion period. To compensate for losses of native species induced by invasions of non-native species, native species reintroductions might be an appropriate tool. For this, we further suggested and discussed a novel approach that predicts the outcome of arising interactions by superimposing stable isotope data from alternative sources to better estimate the success of native species´ reintroductions.

## Introduction

The ecological consequences of aquatic invasions have gained substantial attention in recent decades^1,2^. Whilst previous research has addressed species-specific invader impacts on native species^3–5^, ecosystems^6,7^, ecosystem services^8^ or mechanisms underlying invasions^9–11^, attention is now shifting towards impacts on entire communities and complex species interactions^6,12–14^.

In the management of aquatic invasions, tools commonly applied to prevent species introductions include the development and application of risk assessment tools^15–17^. These are usually reliant on extrapolating data from a species’ native range or from other introduced populations to identify potential threats and ecological impacts that would arise in a new area^18,19^. Alternative techniques to predict impacts using modelling^20^ or experimental approaches^21–23^ have also been proposed. However, the extent to which these approaches can scale up to predict impacts in more complex systems can have high context dependency^24,25^. More recent methods, such as stable isotope analysis (SIA^26^), provide new possibilities to investigate aquatic invasion risks and their associated impacts. This is especially pertinent in invaded communities where there is a high level of complexity in species interactions, which in turn could potentially lead to novel, but often less conspicuous impacts on native species and aspects of their habitats^27,28^.

Stable isotope analyses (SIA) provide long-term and time-mediated information on consumed trophic resources^29,30^ and are used to describe quantitatively the trophic relationships occurring among organisms, highlighting potential diet overlap and feeding competition between species^31^ and estimating the proportion of different preys in the diet^32^. Accordingly, it can be used to investigate the impact of invasive species on native ones^4,26^ and on local communities^27,33^. SIA is based on predictable changes in nitrogen (δ^15^N) and carbon (δ^13^C) isotope ratios between consumers and their food sources^34^: δ^15^N indicates the trophic position within a food web, while δ^13^C identifies major energy sources. Following the common practice of risk assessments^19^ extrapolating species information from one region onto another^15–17^, we suggest that stable isotopes can be used in a similar fashion.

To test the suitability of stable isotope analysis to better understand invasive species’ impacts on native communities, a simplified study system is required, such as the invaded community of Arreo Lake (Spain). Prior to 1990, various taxa of native fish and invertebrates were present in Arreo Lake; however, they are now considered extirpated or locally extinct^5^. Among these, some are of regional concern: the European eel *Anguilla anguilla* Linnaeus, 1758 (considered extirpated after 2000), the endemic whirligig beetles *Gyrinus* sp. Geoffreoy, 1762 (considered extirpated after 2008), the common tench *Tinca tinca* Linnaeus, 1758 (considered extirpated after 2013), and the white-claw crayfish *Austropotamobius pallipes* Lereboullet, 1858 (considered extirpated after 2014). Moreover, *A. anguilla* and *A. pallipes* are both included in the IUCN red list risk categories as “critically endangered” and “endangered”, respectively^35,36^. These extirpations were mainly attributed to subsequent introductions of various non-native species which out-competed or predated native species^37,38^. The protected area of Arreo Lake was not utilized for angling nor changed in terms of water quality^33,38^, but non-native fish and crayfish species introductions likely relate to introductions from illegal anglers or the live-bait pathway (Table 1). The resultant non-native species community is characterised by high linkage density and connectance in the food-web due to its strong trophic interactions and predator-prey relationships^5,33^. In particular, in the last decade, the community and habitat of Arreo Lake has substantially changed through the loss of native fishes and macrophytes. Particularly the presence of the crayfish *Procambarus clarkii* has led to disruptions of the habitat and the native flora, leading to the dominance of *Phragmites australis* and *Cladium mariscus*; both non-native plants^5^.

**Table 1:** Non-native species present in Arreo Lake, showing the year of its first detection and the most likely pathway.

**Group**	**Species**	**First detected**	**Reference**	**Pathways**
**Non-native species**				
Fish	*Micropterus salmoides*	1994	^39^	Angling
	*Lepomis gibbosus*	2005	^38^	
	*Cyprinus carpio*	2010	^38^	
Crayfish	*Procambarus clarkii*	1998	^38^	Aquaculture
Plants	*Phragmites australis*	1998	^5^	Unknown
	*Cladium mariscus*	1998	^5^	

**Group**	**Species**	**Extirpation date**	**Reference**	**Pathways**
**Native species**				
Fish	*Anguilla anguilla*	2000	^5^	Not applicable
	*Tinca tinca*	2013	^5^	Not applicable
Crayfish	*Austropotamobius pallipes*	2014	^5^	Not applicable
Insects	*Gyrinus* sp.	2008	^5^	Not applicable

As control efforts in Arreo Lake failed to eradicate non-native species and to restore populations of native species, new possibilities to control the non-native species have been investigated. Haubrock et al.^33^ explored the effectiveness of reintroducing a once native predator, the European eel *Anguilla anguilla* (Linnaeus, 1758) as a bio-control agent by combining standardized stable isotope data from two sources (i.e. the target community Arreo Lake and the target species *A. anguilla* from a German Lake), dietary analyses and literature reviews. As a result, that particular study highlighted that the reintroduction of eels could affect species in lower trophic positions, while the eel itself could potentially be preyed upon by the introduced top predator *Micropterus salmoides*, limiting the efficacy of this management effort.

In Arreo Lake, as well as many other aquatic ecosystems, the exact causes of native species population extirpations have not yet been fully identified, preventing the design and implementation of appropriate restoration actions. To address this, the aim of our study was to develop and present a new methodological approach that projects stable isotope data onto the isotopic relationships of a focal community. Such an approach could potentially be used in two different scenarios:

- It could be applied retrospectively to reconstruct impacts of non-native species on extinct native species, with some caveats regarding uncertainties. Thus, by theoretically introducing stable isotope data from a species present in community A into community B where it is extinct, this approach could help understand the extent to which non-native species were responsible for these extinction of native species versus other possible explanations (e.g. changes in the abiotic components), and the mechanisms involved (e.g. predation or the adverse effects of increased inter-specific competition).
- It could also be used to predict potentially arising interactions (competition, predation, etc.) to eventually evaluate the extinction risk of native species in invaded ecosystems or ecosystems at risk of being invaded. This includes a new option for water managers to use this approach to better predict reintroduction success of native species in invaded ecosystems.

The simplicity of the current non-native species community in Arreo Lake provides an ideal model system for the described approach of using foreign stable isotope data (i.e. from a different community) from once native but today extirpated species with the aim of investigating possible reasons of extirpations and the probability of successful reintroduction attempts. The results obtained from this novel methodological approach will lay the groundwork for future studies utilizing stable isotopes in explanatory ways to depict antecedent and prospective species interactions.

## Results

**Figure 1.**
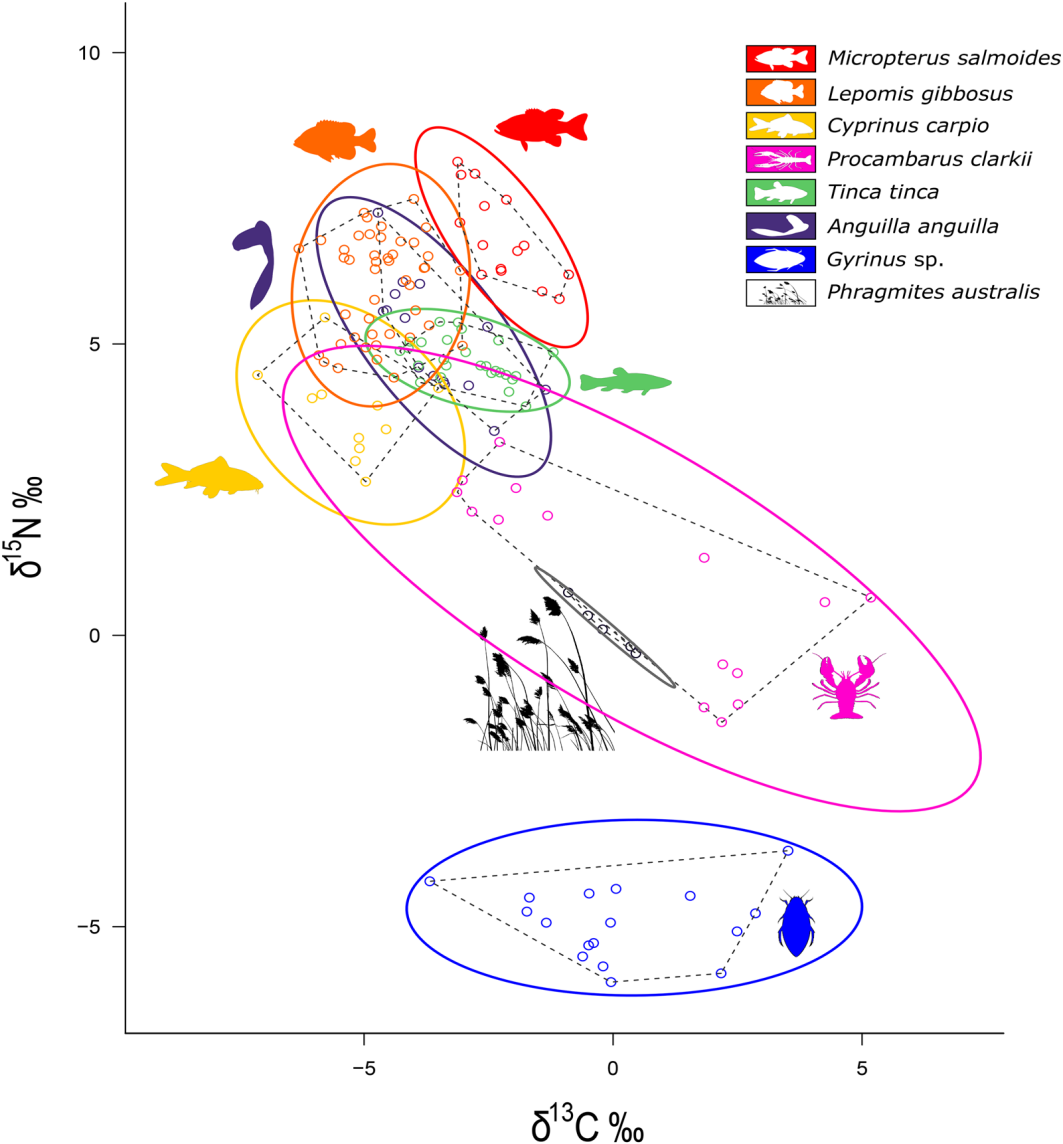
Isotopic niches of non-native species present in Arreo Lake and the as present assumed native *T. tinca and Gyrinus* sp.. Solid line = 95% Standard Ellipse Area (SEAb); dashed line = Total Hull Area (TA); blue: *Gyrinus* sp.; brown: *Phragmites australis*; pink: *Procambarus clarkii*; yellow: *Cyprinus carpio*; orange: *Lepomis gibbosus*; green: *Tinca tinca*; purple: *Anguilla anguilla*; red: *Micropterus salmoides*.

The inclusion of *T. tinca* within the species community had the effect of decreasing the trophic distance among the species while not increasing the overall niche area (see CD and NND; Table 2). The inclusion of *Gyrinus* sp. and *A. anguilla*, however, led to a substantial increase in the community’s isotopic niche area (Table 2). The isotopic niche of *T*. *tinca* was small compared with other fish species. Yet, *T. tinca* occupied a higher trophic position than the non-native *C. carpio*, its closest ecological analogue in the lake (Table 2; Fig. 1). *Anguilla anguilla* occupied a predatory position, expressing an isotopic niche similar to those of *L. gibbosus* and *M. salmoides*. Conversely, *Gyrinus* sp. was predicted to occupy the lowest trophic position of all investigated species, whilst also having a relatively large isotopic niche (Table 2; Fig. 1). However, the carbon range of the non-native macroinvertebrate *P. clarkii* was greater than for *Gyrinus* sp., while *T. tinca* expressed a lower carbon range than *C. carpio* (Table 2).

**Table 2:** Layman metrics for all species and the assumed present *T. tinca, Gyrinus* sp.*, and A. anguilla* as well as the community with and without them.

Species	Trophic position	Nitrogen range [δ^15^N]	Carbon range [δ^13^C]	Total hill area	CD	NND	SDNND	SEAc	SEAb
**Communities**									
Community****	–	9.6	12.3	53.78	3.19	0.43	0.35	–	
Community_T*	–	14.6	12.3	53.78	2.52	0.34	0.31	–	
Community_G**	–	14.1	12.3	104.41	4.65	0.46	0.39	–	
Community_A***	–	14.1	12.3	106.08	4.07	0.36	0.32	–	
**Non-native species**									
*M. salmoides*	4.0	2.3	2.2	2.50	0.91	0.31	0.17	1.25	7.49
*L. gibbosus*	3.5	3.8	3.3	4.95	1.19	0.48	0.43	2.23	13.35
*C. carpio*	3.1	2.9	3.6	5.1	1.04	0.54	0.49	2.42	14.52
*P. clarkii*	2.3	4.8	8.3	16.15	3.07	0.63	0.41	10.17	60.95
*P. australis*	Baseline	1.0	1.4	0.11	0.56	0.33	0.17	0.14	0.84
**Native species**									
*T. tinca*	3.4	1.5	3.1	2.98	0.82	0.22	0.20	3.85	23.08
*Gyrinus* sp.	2.1	2.3	7.2	9.44	1.50	0.57	0.49	0.89	5.33
*A. anguilla*	3.8	3.1	3.3	7.50	1.03	0.25	0.17	1.98	11.88

* CD* mean distance to centroid,* NND* mean nearest neighbour distance,* SDNND* standard deviation of the nearest neighbour distance,* SEAc* corrected Standard Ellipse Area considering 40 % data coverage,* SEAb* corrected Standard Ellipse Area considering 95 % data coverage.

^*^Under consideration of *T. tinca* being present in Arreo Lake.

^**^Under consideration of *Gyrinus* sp. being present in Arreo Lake.

^***^Under consideration of *A. anguilla* being present in Arreo Lake.

^****^Under consideration of neither *T. tinca* nor *Gyrinus* sp. being present in Arreo Lake.

**Figure 2.**
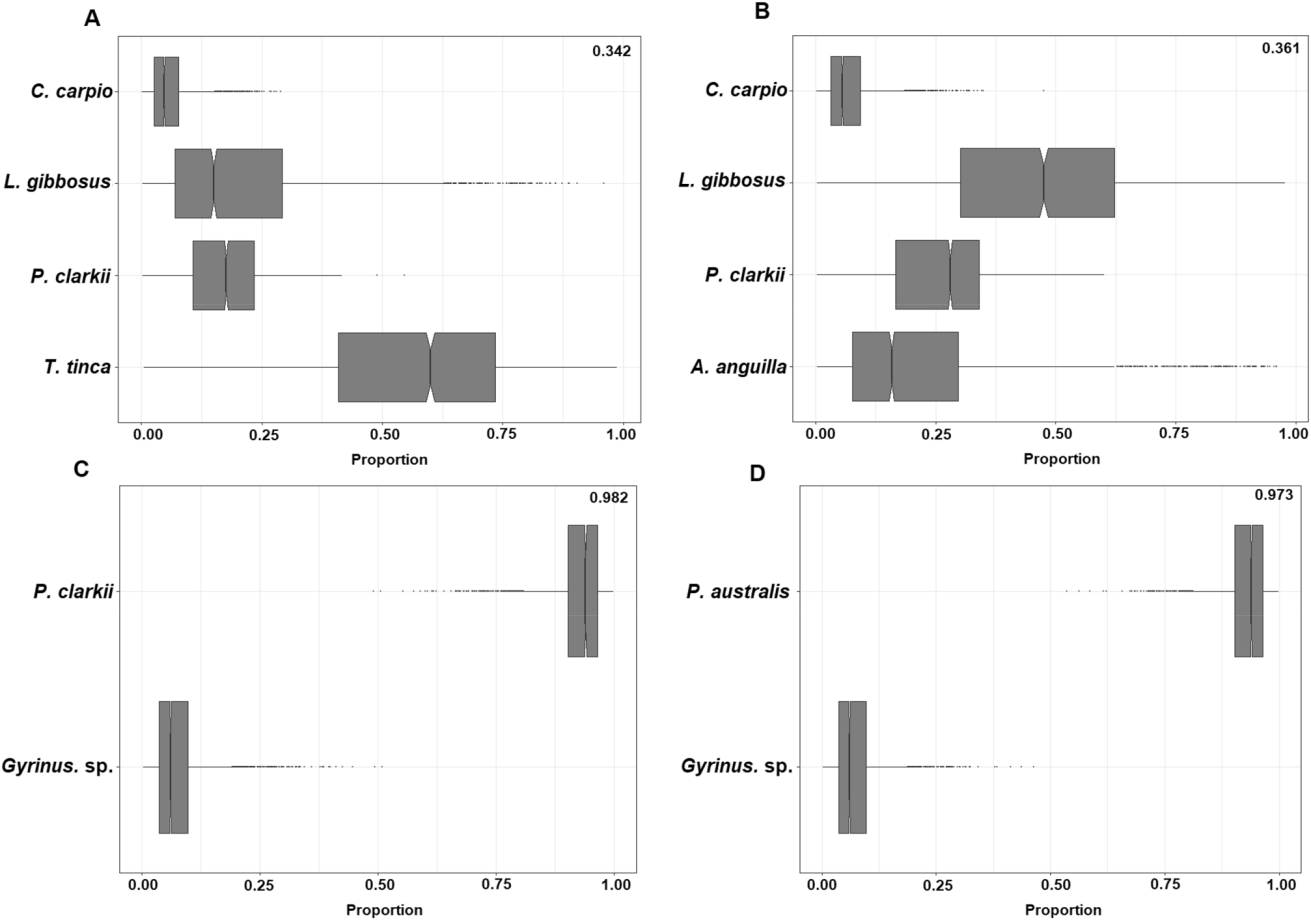
Mixing models estimated for the respective predatory species: (**A**) *Micropterus salmoides* and, *Tinca tinca* as potentially available prey; (**B**) *Micropterus salmoides* under the assumption of present *A. anguilla*; (**C**) *Lepomis gibbosus* assuming that *Gyrinus* sp. is present in Arreo Lake; and (**D**) *Procambarus clarkii* under the assumption of present *Gyrinus* sp.. Numbers in the upper right corner indicate the estimated probability of the presented prey contribution to the respective predators’ isotopic level.

The predicted and actual isotopic niches (as SEAb) of the species in the lake revealed that when *T. tinca* was included in the analyses, shared dietary resources overlap would occur with *L. gibbosus* and *P. clarkii*, but also partially with *C. carpio* (Fig. 1; Table 3). It was also estimated that *M. salmoides* would occupy a distinct isotopic niche to *T. tinca* and was positioned higher in the food web (Fig. 1; Table 2). In contrast to *T. tinca*, *A. anguilla* expressed high overlap with *L. gibbosus* and *C. carpio* while *Gyrinus* sp. was projected to occupy a distinct isotopic niche at a low position in the food web (Fig. 1). It did not share isotopic space with any other species nor did it express the potential to occur within another species isotopic niche (Table 3). The applied mixing models predicted that, based on these isotopic values, *M. salmoides* had a relatively high probability of predating on *T. tinca* (Fig ure 2a), but not on *A. anguilla* (Fig. 2b). For *Gyrinus* sp., the mixing models predicted that neither *L. gibbosus* nor *P. clarkii* expressed the likeliness to be an active predator (Fig. 2c,d).

**Table 3:** SEAb overlap among non-native species in Arreo Lake and *T. tinca, A. anguilla, and* *Gyrinus* sp. as well as the probability of *t*hese to occur in the niche of respective non-native species.

	SEAb overlap	% isotopic niche overlap (%)	Probability of overlap (%)
***T. tinca***			
Non-native species			
*M. salmoides*	0.01	0.1	2.9
*L. gibbosus*	4.72	33.8	89.4
*C. carpio*	1.36	4.3	12.6
*P. clarkii*	2.75	7.4	32.6
***A. anguilla***			
Species			
*M. salmoides*	0.82	4.4	2.9
*L. gibbosus*	7.47	42.0	70.4
*C. carpio*	3.88	17.2	21.0
*P. clarkii*	2.59	3.7	12.1
***Gyrinus* sp.**			
Species			
*M. salmoides*	0.00	<0.1	0
*L. gibbosus*	0.00	<0.1	0
*C. carpio*	0.00	<0.1	0
*P. clarkii*	0.00	<0.1	0

## Discussion

In our study, we used highly variable stable isotope data, making our analyses subject to some degree of uncertainty. However, our main aim was to provide a novel theoretical framework to help predict trophic interactions that are no more ongoing due to species extirpations. Combining stable isotope data from different locations relies on the assumption that the trophic niche of a species is conservative^40^. Hence, to make trophic levels comparable, it is essential to scale isotopic data with the local baseline, which can show substantial spatial variation^34^. Second, although the trophic niche could vary (especially for generalist species) depending on the availability of different resources and the community composition or structure in different areas, the approach used here follows the thinking-scheme of all existing risk assessments made for potentially invasive species, i.e. the assumption that it is valid to project species’ information (e.g. impacts and behavioural or biological traits) from one area onto another^16,19^. Currently used risk assessment protocols include not only a value for the species-specific risk or impact potential, but also a ‘certainty’ statement (e.g. low, medium, and high^16^). In our approach, it must be considered that the proposed method and risk assessment method focuses only on potentially arising biological interactions, thus neglecting the potential of abiotic changes. Hence, a degree of ‘certainty’ can be obtained *a priori*, by utilising stable isotope data from the most similar communities available in terms of species composition (i.e. potential prey populations), structure (i.e. potential competitors and predators), as well as climatic regions, ecosystem type and habitat carrying capacity. Moreover, the present approach can be replicated for various scenarios if the ecosystem from which a species’ isotope information is extracted has data from a common baseline in the target community. Indeed, this has now been tested successfully both in the present study and in an assessment of the effects of species re-introductions^5^. However, the method can only be considered reliable if biological drivers that could have caused the extirpations of native species are considered with all possible contributing species, and in the absence of abiotic factors. Using more complex systems will likely add higher uncertainties to the estimated results but will still indicate the potential for occurring niche overlap and thus, competition. However, as all risk assessments cannot be free of projected assumptions which may or may not hold true within complex ecosystems due to diverse and unpredictable species interactions, this methodology provides probabilistic estimates, which will correlate with the degree of uncertainty.

### Methodological limitations

A notable downside of this approach is the availability of stable isotope data for the relevant species, as for three species only one set of suitable data and for another species no suitable data were found. As a result, data availability can be seen as a key component, while the data from both community and investigated species, ought to be robust. However, even if stable isotope data for target species are found, the comparability to the target community requires testing in relation to the similarity of a common stable isotope baseline, such as filter feeders and primary producers. Without such a comparable baseline, data usability will be characterized by a considerable degree of uncertainty. Moreover, the lack of such comparable baselines as well as the continuous change within ecosystems leads to ongoing variation on trophic niches of studied species, an aspect that needs to be considered in the interpretation of estimated results. However, the greatest obstacle will be the limited availability of stable isotope data. We therefore echo the call of Pauli et al.^41,42^ for the importance of a centralized repository for stable isotope data. Another aspect that has to be mentioned is the fact that the tissue samples collected from Arreo Lake are not influenced by any of the native species that we are superimposing on its food web. Therefore the proportion of the diet that the natives contribute to each predator analysed is in reality 0, while we assume that the simplified food web has a similar trophic height to that of the native condition, so that when we reintroduce the values for the native species they will lie in the same isotopic niche relative to these invasive species as when both were present in the ecosystem.

### Biotic pressure and extinction risks

In scenario *(a)*, where we retrospectively estimated that the introduced *M. salmoides* was a potential predator of *T. tinca,* our findings support other studies on the deleterious impact of invasive *M. salmoides* populations^43–46^. In addition, we identified the potential for dietary resource overlap among *T. tinca* and *L. gibbosus* as well as *C. carpio*. Recent work by Almeida et al.^47^ suggests that *L. gibbosus* is aggressive towards native Iberian species and hence, potentially also *T. tinca*. Moreover, considering the presence of *P. clarkii* and *L. gibbosus*, as well as information of their invasion histories, it is possible that the resource overlap among these species and *T. tinca* resulted in increased competition, due to the likely reduction of algae (due to *P. clarkii*) and macroinvertebrate prey due to *L. gibbosus*^48^ and *P. clarkii*^49^. Another aspect that has to be considered is the possibility of biotic changes and associated indirect effects. Indeed, the presence of *L. gibbosus* and *P. clarkii* could have reduced potential prey for *T. tinca* (especially molluscs) and aquatic vegetation (amplified by the positive effect of *C. carpio* on turbidity^50^) in shallow areas needed for reproduction^51^.

In the case of *Gyrinus* sp., mixing models indicated that neither *P. clarkii* nor *L. gibbosus* are a major predator, indicating a potential problem with the used baseline while simultaneously indicating the belonging of *Gyrinus* sp. to a non-aquatic trophic pathway. However, especially in very shallow (depths of few cm) but vegetated zones, (i.e. the habitat occupied by both species^52,53^, predation by both *P. clarkii* and *L. gibbosus* on especially larval stages of *Gyrinus* sp. is possible^48,54^. It should be noted that *L. gibbosus* commonly exerts a more bottom and water column orientated feeding activity^55^. Additionally, as the flying insect abundance potentially decreased due to the decreasing abundance of native flora and increasing abundance of *P. australis*^56,57^, a lack of potential prey might have aided the decline of *Gyrinus* sp.^58,59^. As a result, considering the high abundance of invasive species in Arreo Lake in the past, possible substantial stressors were (a) feeding pressure, (b) competition for declining resources, as well as (c) bioengineering activities that alter the suitability for reproduction.

Multiple stressors contributing to the demise of native species have been reported for communities of different sizes and structures^60,61^. With the proposed approach, we could show clear indications of biotic interactions, mostly predation and competition, both potentially affecting the native species. While a considerable uncertainty must be accepted, the observed outcomes also indicate direct (e.g. through resource overlap) and indirect effects (e.g. the demise of potential prey) that could have led to the demise of native species. Accordingly, especially the habitat engineering activity of *P. clarkii*^62^ and *C. carpio*^63,64^, thus also the changing vegetation, could have led to substantial alterations that negatively affected suitable reproduction habitat for *T. tinca* and decreased prey abundance of *Gyrinus* sp.. As such, in the case of *T. tinca* and *Gyrinus* sp., it is probable that a combination of direct and indirect biotic stressors led to the demise of both species.

### Evaluating reintroduction opportunities

Management efforts to control non-native species are common practice^65,66^ and should be considered prior to any reintroduction attempts^67,68^. Such management actions aim towards the recovery of native species populations and restoration of habitats^67^. In Arreo Lake, these efforts already led to a considerable decline in non-native species abundance^5^. Using our proposed approach from scenario *(b)*, we argue that in the case of *Gyrinus* sp., this would mean a decrease in the abundance of the invasive species *P. clarkii* and *L. gibbosus*, but also a recovery of natural vegetation and consequently, terrestrial insects in the proximity of Arreo Lake that could be predated by *Gyrinus* sp.. Additionally, the extinction of *Gyrinus* sp. likely led to a shortening of the trophic chain, leading to a simplification of the community structure, so that its recovery could favour other species. For *T. tinca*, recovery could be obtained through the removal of its main predator *M. salmoides*. Furthermore, its recovery could be facilitated with the retrieval of the aquatic habitat structure that was bioengineered by *P. clarkii* but is needed for reproduction. Reducing all biotic stressors (competition, predation, lack of suitable prey or habitat required for reproduction, etc.) will improve the chances of a successful recovery of native species and should therefore be the main aim for future management efforts in Arreo Lake. Being a protected habitat, angling activities in Arreo Lake are nowadays strictly forbidden, however, declining populations of *A. anguilla* are a pressing issue^69,70^. The reintroduction and use of *A. anguilla* as biocontrol agent was proposed by Benndorf^71^ and Aquiloni et al.^72^. Haubrock et al.^33^ tested the use of stable isotopes to predict the impact of introducing predatory eels on the non-native species community in Arreo Lake as described in scenario *(b)*. In that study, they indicated that despite seasonal variation in feeding activity, *A. anguilla* would predate both *L. gibbosus* and *P. clarkii*. Nevertheless, a moderate predation by *M. salmoides* on eels was found to be likely, in concordance with the feeding behaviour of *M. salmoides*^73^. Additionally, it is likely that *M. salmoides* might function as a competitor for eels^74^, as indicated by its wide feeding behaviour and wide trophic niche^75^. Accordingly, other possibilities that have led to the extinction of *A. anguilla* must be considered, as such, overfishing^69^ and the closure of the reproductive migration through dams^76,77^. Consequently, the recovery of *A. anguilla* is strictly dependent also by the construction of comeback ladders.

### Conclusion

Our approach can be used from both a scientific and a management perspective to inform risk-based management programmes and by contributing to knowledge on invasion impact assessments. Particularly by being aware of potential biological drivers of impact, it helps unravelling the mechanisms behind the declines of native species, while future reintroduction projects may profit from such insights by better estimating the success rate of native species to recover. The application of stable isotope mixing models and bi-plots allow predictions of the importance of biotic stressors and, from a management perspective, enables practical actions. Further research should examine whether the reduction of non-native populations in aquatic systems could be sufficient to enable the recovery of endangered target species, by mediating predation and interspecific competition, as indicated by the presented stable isotopes approach. Furthermore, this method could potentially be applied to other more complex communities and ecosystems to aid in invasive species prioritization by managers or inform control efforts to enable the recovery of native or endangered species. It also provides potential for testing aspects of relevant trophic niche theory relating to biological invasions (e.g.^78,79^), promoting the use of extant data rather than designing and completing new experiments.

## Methods

### Study site

Arreo Lake covers 1.36 km^2^ and is situated 655 m above sea level. As part of the Ebro river basin, it is considered the only natural lake in Basque Country, Northern Spain^37^. The southern areas of the lake are shallow^80^, while on the north side a basin with a steep slope and a depth of 24 m is located. Arreo Lake is peculiar, as it is naturally fed by hypersaline water from the diapiric substratum (1307–1608 µS/cm^37,81^), which has resulted in the emergence of a unique ecological community. Indeed, Arreo Lake has previously provided habitat to various endemic invertebrate species and a unique species assemblage^82^. However, today, Arreo Lake hosts a very simplified community composed by only non-native species, namely three fish species and one crayfish (Table 1). Similarly, the native flora, of which six species were considered endangered, has been entirely replaced by dense populations of the non-native macrophyte *Phragmites australis* and the less abundant *Cladium mariscus*^5^.

### Data collection

To test our approach, stable isotope data from the currently existing community in Arreo Lake and the extirpated native species of interest were collected. For the six non-native species (Table 1), stable isotope data (nitrogen [δ^15^N] and carbon [δ^13^C]) were collected recently (September 2017^33^). We selected the following four native species that were recently extirpated from Arreo Lake, namely *A. anguilla, T. tinca, A. pallipes* and *Gyrinus* sp. (LIFE TREMEDAL project LIFE11 NAT/ES/ 000707; ES2110007). As these species were extirpated, their isotope data could not be collected from Arreo Lake nor individuals from the lake were present in museum collections and therefore, was retrieved from the literature. To identify such potentially suitable stable isotope data for these extirpated native species, we used the ISI Web of Science platform (https://webofknowledge.com/), Google Scholar (https://scholar.google.com/), the Google search engine (https://www.google.com/) and through contacts with experts. Hence, data availability was considered as the first criterion, followed by data suitability. Documents identified as potentially relevant were thoroughly assessed. Then, it was ensured that the tissue type for fish and crayfish (caudal muscle) and plants (leaves) was consistent, while trying to obtain data from the same seasonal period.

When further selecting data sources for the extirpated native species to be superimposed on the extant non-native species community, a common baseline organism (i.e. a species that can be used as a reference point to estimate the trophic position of species at higher trophic levels in the food web) must occur in both ecosystems. The suitability and comparability of the baseline organism was determined on the basis of similar ranges (95% confidence interval) in δ^15^N and δ^13^C in both ecosystems. If available, this baseline was then used to standardize the stable isotope data from the community and target species. The data identified as suitable is listed in Table 4. For *T. tinca* and *A. anguilla*, this was accomplished using the macrophyte *P. australis* from either respective community. Note that macrophytes were chosen as the isotopic baseline due to the lack of other commonly used baselines, such as gastropods or filter feeders^81^, in Arreo Lake. The use of macrophyte isotope signatures can be problematic, as this group tends to demonstrate variability between seasons, watersheds, nutrient levels, depth, and even tissue in combination with relatively rapid turnovers. However, we argue that the use of pooled samples, i.e. the combination of leaf tissue from minimum five individual plants per site into one sample in combination with a sufficiently large sample that covers the entire ecosystem while expressing inherently low spatial and temporal variability can be sufficient for a proximate analysis of communities. Further, *P. australis* is less prone to express highly spatially or temporally variable trophic shifts as variations are mostly linked to ambient changes in salinity which are generally not strongly fluctuating in isolated lakes^83,84^. Hence, it should be sufficient for the momentary analysis and first step in this novel application as proposed here.

**Table 4:** Origin and ranges of finally considered stable isotope data after standardization by subtracting the communities’ respective baseline.

Species	Country	Location	Stable isotope data origin	n	Mean δ^15^N + range [‰]	Mean δ^13^C + range [‰]	Tissue type
*Tinca tinca*	England	Small ponds	^88^	26	4.7 (3.9–5.4)	− 2.9 (− 4.3 to − 1.2)	Caudal muscle
*Anguilla anguilla*	Germany	Lake ecosystem Großer Vätersee	^89^	45	6.0 (4.4–7.5)	− 4.7 (− 6.3 to − 3.0)	Caudal muscle
*Austropotamobius pallipes*	–	–	–	–	–	–	Tail muscle
*Gyrinus* sp.	Canada	Experimental Lake area in Western Ontario	^85^	17	− 4.9 (− 6.0 to − 3.8)	0.2 (− 3.6 to 3.6)	Whole organism
*Micropterus salmoides*	Spain	Arreo Lake in Basque country	^33^	15	6.8 (5.8–7.9)	− 2.2 (− 3.1 to − 0.9)	Caudal muscle
*Cyprinus carpio*				11	3.8 (2.6–5.5)	− 5.3 (− 7.1 to − 3.5)	Caudal muscle
*Lepomis gibbosus*				15	5.1 (3.5–7.3)	− 3.6 (− 4.7 to 1.4)	Caudal muscle
*Procambarus clarkii*				15	1.0 (− 1.5 to 3.3)	0.4 (− 3.1 to 5.2)	Tail muscle
*Phragmites australis*				5	0.0 (− 0.4 to 0.6)	− 0.0 (− 0.7 to 0.6)	5 x 5 pooled leaf samples
*Cladium mariscus*				Not sampled			

For *Gyrinus* sp. only one potentially suitable study was identified^85^, as the number of specimens reported from other studies (e.g.^86^; n = 3) was too low. Despite the lack of any comparable baseline organisms (e.g. macrophytes) or other occurring species between both Kullman et al.^85^ and Arreo Lake, comparability of stable isotope data was accepted under the assumption that species in lower trophic positions like *Gyrinus* sp. express lower degrees of niche variability among different populations due to limited isotopic variability in their potential prey compared to species in higher trophic positions^87^. In this case, we selected the standardization with the Arreo Lake macrophyte, assuming it to be a valid alternative. In the case of *A. pallipes*, no stable isotope data was found, likely due to its protected status and limited distribution. This was also true for the ecologically similar and closely related stone crayfish *A. torrentium*.

Suitable stable isotope data were complemented with information on the biology, ecology and dietary data of the respective species (Supplement 1). Due to lack of suitable stable isotope data for *A. pallipes*, information on dietary preferences and impacts were reviewed only for *Gyrinus* sp., *T. tinca, A. anguilla* and the non-native species present in Arreo Lake. For these species, results from the literature reviews were combined with stable isotope data for the non-native species from Arreo Lake to reconstruct their interactions and thus their ecological impacts. All stable isotope data that were used are presented in Supplement 2.

### Data analysis

Standardization of isotope data (δ^15^N and δ^13^C) was performed for all species in Arreo Lake as well as for the values extracted for extirpated native species according to Haubrock et al.^33^ by subtracting the populations specific baselines’ mean value for δ^15^N and δ^13^C, respectively, to make isotopic data comparable (Table 2). Then, both data arrays of the target community and the introduced species were combined.

To compare ecological roles of the once native *T. tinca* and *Gyrinus* sp. in contrast to the non-native species present in Arreo Lake, the trophic position for each species was estimated. This was done using the R package “tRophicPosition”, which uses Markov Chain Monte Carlo simulations^90^. Because trophic discrimination factors (TDFs) were not available for all species, we included simulated tissue discrimination factors from Post^34^ using the R function ‘*simulateTDF.tRophicPosition*’^90^.

To quantify isotopic niches and identify changes in the isotopic community structure following the respective decline of *T. tinca* and *Gyrinus* sp., Layman’s metrics were calculated^91,92^ for each species individually as well as for the community with and without it. Layman’s metrics describe the isotopic niche dimensions of a species or a community, while the isotopic niche is the trophic space of a species which is affected by e.g. growth and metabolism or isotopic turnover and defined by individual points in a two-dimension isotopic space^91^.

Moreover, the Bayesian standardized ellipse areas (SEAc: alpha = 0.4; SEAb: alpha = 0.95), which encompass a sampled population in a δ^15^N/δ^13^C bi-plot space and thus serves as measures of core niche space, as well as the proportion of overlap in the isotopic niches^92^, were calculated in the R package “SIBER”^26^. These estimated the isotopic niche overlaps among the species currently present in Arreo Lake and the extirpated *T. tinca* and *Gyrinus* sp., therefore indicating potentially competitive interactions. Additionally, the R package “NicheRover” was used to calculate the directional pairwise probability of either targeted species occurring within the niche of other species from Arreo Lake. This approach utilizes a Monte Carlo estimation (chain length: 20.000 steps), computing the probability of the niche of species ‘A’ to overlap onto the niche of species ‘B’^93^, therefore highlighting potentially occurring niche overlap.

Lastly, to investigate predation as a cause of extinction, mixing models as part of the R package “simmr”^32^ were employed. The application of mixing models relies on the use of dual plot graphs for δ^15^N and δ^13^C, enabling assumptions on probable prey sources and combinations of prey contributing to the diet of consumers. Hence, mixing models were employed for predatory species that could consume native species on the assumption that predation occurs on lower trophic levels due to δ^15^N increments across trophic levels. Only one native species was included in the community at once to determine the pathway that is the major source of energy as both *T. tinca* and *Gyrinus* sp. belong to different energy pathways (i.e. terrestrial versus aquatic). Including both together could lead to a meaningless confounding effect. Further, mixing models were presented with the estimated probability of each scenario, i.e. the likeliness of the identified prey contributing to the respective predators’ isotopic level.

## Supplementary information

Supplementary Information 1.
