# Supplementary material for: Using stable isotopes to analyse extinction risks and reintroduction opportunities of native species in invaded ecosystems
Source: Sci Rep. 2020 Dec 10;10:21576. doi: 10.1038/s41598-020-78328-9 (PMC7728764; doi:10.1038/s41598-020-78328-9)
Supplement: Supplementary file 1 — Supplementary Information 1. [file 41598_2020_78328_MOESM1_ESM.docx]

**Using stable isotopes to analyse extinction risks and reintroduction opportunities of native species in invaded ecosystems**

**Short title: Isotope Impact Projection**

**Phillip J. Haubrock| Paride Balzani|J. Robert Britton| Peter Haase**

**Supplement 1**

| **Species** | **Literature used containing valuable information** |
| --- | --- |
| *Tinca tinca* | Busst et al., 2015; Britton et al., 2018; Pompei et al. 2012; Froese, R., & Pauly, D. (2010); |
| *Anguilla anguilla* | Froese, R., & Pauly, D. (2010); Lammens et al. 1985; Costa et al. 1992; Dörner et al., 2009;  Benndorf (1995); Aquiloni et al. 2010  Barak and Mason 1992; Tesch 1999 |
| *Gyrinus* sp. | Chambers et al., 1999; Gratton & Denno, 2005; |
| *Micropterus salmoides* | Gratwicke & Marshall 2001; Maezono and Miyashita 2003; Yonekura, Kita, & Yie. 2004; Maezono, Kobayashi, Kusahara, & Miyashita 2005; Froese, R., & Pauly, D. (2010); Schiphouwer et al. 2017; Costantini et al. 2018; McCord, 2000 |
| *Cyprinus carpio* | Fletscher et al., 1985; Froese, R., & Pauly, D. (2010); Britton et al. 2007; Anton-Pardo et al. 2014 |
| *Lepomis gibbosus* | Almeida et al., (2009); Jastrebski & Robinson, 2004; Wolfram-Wais et al., 1999; Froese, R., & Pauly, D. (2010) |
| *Procambarus clarkii* | Angeler et al., 2001; Oficialdegui et al., 2020; Barbaresi et al 2004; Gherardi & Barbaresi, 2008 |
| *Phragmites australus* | Fell et al., 1998; Bedford& Powell, 2005; Chambers et al., 1999; Gratton & Denno, 2005 |

**Supplement 2**

| ***Species*** | **Origin** | **d15N** | **D13C** |
| --- | --- | --- | --- |
| *C. carpio* | Arreo Lake | 13.857 | -34.384 |
| *C. carpio* | Arreo Lake | 11.936 | -33.158 |
| *C. carpio* | Arreo Lake | 12.644 | -32.105 |
| *C. carpio* | Arreo Lake | 12.868 | -35.739 |
| *C. carpio* | Arreo Lake | 12.470 | -34.641 |
| *C. carpio* | Arreo Lake | 11.790 | -33.706 |
| *C. carpio* | Arreo Lake | 11.037 | -33.567 |
| *C. carpio* | Arreo Lake | 12.535 | -34.450 |
| *C. carpio* | Arreo Lake | 11.391 | -33.774 |
| *C. carpio* | Arreo Lake | 12.343 | -33.330 |
| *C. carpio* | Arreo Lake | 11.611 | -33.694 |
| *L. gibbosus* | Arreo Lake | 13.694 | -31.120 |
| *L. gibbosus* | Arreo Lake | 13.959 | -33.214 |
| *L. gibbosus* | Arreo Lake | 11.908 | -30.984 |
| *L. gibbosus* | Arreo Lake | 12.996 | -32.502 |
| *L. gibbosus* | Arreo Lake | 12.616 | -29.956 |
| *L. gibbosus* | Arreo Lake | 13.979 | -33.148 |
| *L. gibbosus* | Arreo Lake | 15.654 | -33.321 |
| *L. gibbosus* | Arreo Lake | 12.853 | -32.206 |
| *L. gibbosus* | Arreo Lake | 12.754 | -32.005 |
| *L. gibbosus* | Arreo Lake | 12.705 | -31.983 |
| *L. gibbosus* | Arreo Lake | 12.688 | -31.499 |
| *L. gibbosus* | Arreo Lake | 14.433 | -32.480 |
| *L. gibbosus* | Arreo Lake | 13.847 | -32.777 |
| *M. salmoides* | Arreo Lake | 15.089 | -30.386 |
| *M. salmoides* | Arreo Lake | 15.877 | -30.741 |
| *M. salmoides* | Arreo Lake | 14.590 | -31.241 |
| *M. salmoides* | Arreo Lake | 16.305 | -31.652 |
| *M. salmoides* | Arreo Lake | 15.098 | -31.215 |
| *M. salmoides* | Arreo Lake | 14.591 | -29.491 |
| *M. salmoides* | Arreo Lake | 14.690 | -30.853 |
| *M. salmoides* | Arreo Lake | 14.656 | -30.845 |
| *M. salmoides* | Arreo Lake | 14.303 | -30.025 |
| *M. salmoides* | Arreo Lake | 15.483 | -31.676 |
| *M. salmoides* | Arreo Lake | 14.996 | -30.518 |
| *M. salmoides* | Arreo Lake | 14.173 | -29.678 |
| *M. salmoides* | Arreo Lake | 16.322 | -31.373 |
| *M. salmoides* | Arreo Lake | 16.528 | -31.721 |
| *M. salmoides* | Arreo Lake | 15.769 | -31.182 |
| *L. gibbosus* | Arreo Lake | 14.258 | -32.977 |
| *L. gibbosus* | Arreo Lake | 14.465 | -32.819 |
| *P. clarkii* | Arreo Lake | 11.719 | -30.867 |
| *P. calrkii* | Arreo Lake | 10.928 | -30.539 |
| *P. calrkii* | Arreo Lake | 10.524 | -31.424 |
| *P. calrkii* | Arreo Lake | 10.455 | -29.903 |
| *P. calrkii* | Arreo Lake | 9.048 | -23.413 |
| *P. calrkii* | Arreo Lake | 7.216 | -26.080 |
| *P. calrkii* | Arreo Lake | 7.164 | -26.763 |
| *P. calrkii* | Arreo Lake | 6.910 | -26.409 |
| *P. calrkii* | Arreo Lake | 9.731 | -26.759 |
| *P. calrkii* | Arreo Lake | 7.752 | -26.093 |
| *P. clarkii* | Arreo Lake | 8.970 | -24.336 |
| *P. calrkii* | Arreo Lake | 7.901 | -26.387 |
| *P. calrkii* | Arreo Lake | 11.061 | -31.615 |
| *P. calrkii* | Arreo Lake | 10.857 | -31.724 |
| *P. calrkii* | Arreo Lake | 10.385 | -30.890 |
| *P. australis* | Arreo Lake | 7.970 | -27.944 |
| *P. australis* | Arreo Lake | 8.380 | -28.614 |
| *P. australis* | Arreo Lake | 8.088 | -28.050 |
| *P. australis* | Arreo Lake | 9.014 | -29.301 |
| *P. australis* | Arreo Lake | 8.618 | -28.911 |
| *A. anguilla* | Großer Vater See | 7.842 | -23.293 |
| *A. anguilla* | Großer Vater See | 7.62 | -20.959 |
| *A. anguilla* | Großer Vater See | 8.849 | -21.891 |
| *A. anguilla* | Großer Vater See | 8.245 | -22.775 |
| *A. anguilla* | Großer Vater See | 8.141 | -23.756 |
| *A. anguilla* | Großer Vater See | 8.191 | -22.552 |
| *A. anguilla* | Großer Vater See | 7.834 | -22.411 |
| *A. anguilla* | Großer Vater See | 8.612 | -22.888 |
| *A. anguilla* | Großer Vater See | 7.773 | -22.646 |
| *A. anguilla* | Großer Vater See | 8.121 | -22.154 |
| *A. anguilla* | Großer Vater See | 8.222 | -22.997 |
| *A. anguilla* | Großer Vater See | 6.167 | -23.8 |
| *A. anguilla* | Großer Vater See | 6.091 | -22.632 |
| *A. anguilla* | Großer Vater See | 8.531 | -22.828 |
| *A. anguilla* | Großer Vater See | 6.052 | -23.693 |
| *A. anguilla* | Großer Vater See | 6.339 | -22.645 |
| *A. anguilla* | Großer Vater See | 6.359 | -23.355 |
| *A. anguilla* | Großer Vater See | 5.783 | -22.29 |
| *A. anguilla* | Großer Vater See | 5.946 | -23.413 |
| *A. anguilla* | Großer Vater See | 7.896 | -22.314 |
| *A. anguilla* | Großer Vater See | 6.796 | -22.789 |
| *A. anguilla* | Großer Vater See | 6.531 | -22.375 |
| *A. anguilla* | Großer Vater See | 6.301 | -22.854 |
| *A. anguilla* | Großer Vater See | 6.473 | -21.968 |
| *A. anguilla* | Großer Vater See | 6.869 | -23.265 |
| *A. anguilla* | Großer Vater See | 6.68 | -21.583 |
| *A. anguilla* | Großer Vater See | 6.33 | -20.915 |
| *A. anguilla* | Großer Vater See | 7.645 | -22.668 |
| *A. anguilla* | Großer Vater See | 7.787 | -22.389 |
| *A. anguilla* | Großer Vater See | 7.673 | -21.666 |
| *A. anguilla* | Großer Vater See | 8.102 | -21.888 |
| *A. anguilla* | Großer Vater See | 7.997 | -24.2 |
| *A. anguilla* | Großer Vater See | 6.525 | -22.725 |
| *A. anguilla* | Großer Vater See | 7.978 | -23.289 |
| *A. anguilla* | Großer Vater See | 7.456 | -22.048 |
| *A. anguilla* | Großer Vater See | 8.377 | -22.543 |
| *A. anguilla* | Großer Vater See | 6.474 | -23.079 |
| *A. anguilla* | Großer Vater See | 6.938 | -21.856 |
| *A. anguilla* | Großer Vater See | 7.371 | -21.974 |
| *A. anguilla* | Großer Vater See | 8.362 | -21.642 |
| *A. anguilla* | Großer Vater See | 7.664 | -21.685 |
| *A. anguilla* | Großer Vater See | 7.805 | -23.184 |
| *A. anguilla* | Großer Vater See | 7.119 | -22.679 |
| *A. anguilla* | Großer Vater See | 7.882 | -22.679 |
| *A. anguilla* | Großer Vater See | 7.868 | -21.604 |
| *Tinca tinca* | English ponds | 12.76 | -26.52 |
| *Tinca tinca* | English ponds | 13.55 | -27.41 |
| *Tinca tinca* | English ponds | 12.8 | -26.64 |
| *Tinca tinca* | English ponds | 13.66 | -27.48 |
| *Tinca tinca* | English ponds | 13.35 | -26.67 |
| *Tinca tinca* | English ponds | 13.15 | -25.57 |
| *Tinca tinca* | English ponds | 13.36 | -27.7 |
| *Tinca tinca* | English ponds | 12.92 | -27.03 |
| *Tinca tinca* | English ponds | 13.02 | -27.77 |
| *Tinca tinca* | English ponds | 12.92 | -28.28 |
| *Tinca tinca* | English ponds | 12.22 | -26.12 |
| *Tinca tinca* | English ponds | 13.15 | -27.33 |
| *Tinca tinca* | English ponds | 12.79 | -26.81 |
| *Tinca tinca* | English ponds | 13.33 | -28.45 |
| *Tinca tinca* | English ponds | 12.63 | -28.24 |
| *Tinca tinca* | English ponds | 13.32 | -28.22 |
| *Tinca tinca* | English ponds | 13.67 | -27.85 |
| *Tinca tinca* | English ponds | 12.47 | -26.46 |
| *Tinca tinca* | English ponds | 12.74 | -26.32 |
| *Tinca tinca* | English ponds | 13.17 | -28.65 |
| *Tinca tinca* | English ponds | 12.91 | -26.92 |
| *Tinca tinca* | English ponds | 12.92 | -27.72 |
| *Tinca tinca* | English ponds | 12.68 | -26.39 |
| *Tinca tinca* | English ponds | 13.22 | -28.48 |
| *Tinca tinca* | English ponds | 12.83 | -26.73 |
| *Tinca tinca* | English ponds | 12.72 | -27.83 |
| *Gyrinus* sp. | Lake | 3.99 | -28.47 |
| *Gyrinus* sp. | Lake | 2.83 | -29.14 |
| *Gyrinus* sp. | Lake | 3.6 | -30.26 |
| *Gyrinus* sp. | Lake | 3.41 | -29.87 |
| *Gyrinus* sp. | Lake | 3.84 | -30.21 |
| *Gyrinus* sp. | Lake | 3.06 | -28.92 |
| *Gyrinus* sp. | Lake | 4.12 | -32.21 |
| *Gyrinus* sp. | Lake | 3.91 | -29.01 |
| *Gyrinus* sp. | Lake | 3.41 | -28.58 |
| *Gyrinus* sp. | Lake | 3.26 | -26.04 |
| *Gyrinus* sp. | Lake | 2.66 | -28.73 |
| *Gyrinus* sp. | Lake | 3.02 | -29.02 |
| *Gyrinus* sp. | Lake | 4.645 | -25.015 |
| *Gyrinus* sp. | Lake | 2.39 | -28.57 |
| *Gyrinus* sp. | Lake | 2.54 | -26.36 |
| *Gyrinus* sp. | Lake | 3.57 | -25.67 |
| *Gyrinus* sp. | Lake | 3.87 | -26.98 |

**Supplementary’s additional references**

Barak, N. E., & Mason, C. F. (1992) Population density, growth and diet of eels, Anguilla anguilla L.,in two rivers in eastern England. Aquaculture Research 23: 59–70, <https://doi.org/10.1111/j.1365-2109.1992.tb00596.x>

Britton, J.R., Ruiz‐Navarro, A., Verreycken, H. and Amat‐Trigo, F., 2018. Trophic consequences of introduced species: Comparative impacts of increased interspecific versus intraspecific competitive interactions. Functional ecology, 32(2), pp.486-495.

Busst, G. M., Bašić, T., & Britton, J. R. (2015). Stable isotope signatures and trophic‐step fractionation factors of fish tissues collected as non‐lethal surrogates of dorsal muscle. Rapid Communications in Mass Spectrometry, 29(16), 1535-1544.

Costa JL, Assis CA, Almeida PR, Moreira FM, Costa MJ (1992) On the food of the European eel, Anguilla anguilla (L.), in the upper zone of the Tagus estuary, Portugal. Journal of Fish Biology 41: 841–850, <https://doi.org/10.1111/j.1095-8649.1992.tb02712.x>

Lammens EH, Nie HWD, Vijverberg J, Densen WLTV (1985) Resource partitioning and niche shifts of bream (Abramis brama) and eel (Anguilla anguilla) mediated by predation of smelt (Osmerus eperlanus) on Daphnia hyalina. Canadian Journal of Fisheries and Aquatic Sciences 42: 1342–1351, <https://doi.org/10.1139/f85-169>

Tesch FW (1999) Der Aal. Berlin, Parey
